# Robotic total gastrectomy for carcinoma in the remnant stomach: a comparison with laparoscopic total gastrectomy

**DOI:** 10.1093/gastro/goab021

**Published:** 2021-07-19

**Authors:** Zheng-Yan Li, Jia-Jia Liu, Pei-Wu Yu, Yong-Liang Zhao, Yan Shi, Zi-Yan Luo, Bin Wu, Jun-Jie Wang, Feng Qian

**Affiliations:** Department of General Surgery, Center for Minimally Invasive Gastrointestinal Surgery, Southwest Hospital, Third Military Medical University, Chongqing, P. R. China

**Keywords:** robotic gastrectomy, laparoscopic gastrectomy, total gastrectomy, carcinoma in the remnant stomach

## Abstract

**Background:**

Total gastrectomy for carcinoma in the remnant stomach (CRS) remains a technically demanding procedure. Whether robotic surgery is superior, equal, or inferior to laparoscopic surgery in patients with CRS is unclear. This study was designed to compare the efficacy and safety of robotic total gastrectomy (RTG) and laparoscopic total gastrectomy (LTG) for the treatment of CRS.

**Methods:**

In this cohort study, we retrospectively analysed the data from patients who underwent RTG or LTG for CRS at Southwest Hospital (Chongqing, China) between May 2006 and October 2019. The surgical outcomes, post-operative complications, and survival outcomes between the two groups were compared.

**Results:**

Compared with LTG, RTG was associated with similar effective operation time (272.0 vs 297.9 min, *P *=* *0.170), higher total costs (105,967.2 vs 81,629.5 RMB, *P *<* *0.001), and less estimated blood loss (229.2 vs 288.8 mL, *P *=* *0.031). No significant differences were found between the robotic and laparoscopic groups in terms of conversion rate, time to first flatus, time to first soft diet, post-operative hospital stay, post-operative complications, R0 resection rate, and number of retrieved lymph nodes (all *P *>* *0.05). The 3-year disease-free survival and 3-year overall survival rates were comparable between the two groups (65.5% vs 57.5%, *P *=* *0.918; 69.0% vs 60.0%, *P *=* *0.850, respectively).

**Conclusions:**

RTG is a safe and feasible procedure for the treatment of CRS and could serve as an optimal treatment for CRS.

## Introduction

Carcinoma in the remnant stomach (CRS) is defined as gastric cancer (GC) in the remnant stomach after partial gastrectomy for benign disease or GC [1]. The incidence of CRS shows an increasing trend with the early detection of primary GC and improvements in the prognosis of patients [2]. CRS is usually detected at an advanced stage and is associated with a low rate of curative resection and a generally poor prognosis [3, 4]. Total gastrectomy with radical lymphadenectomy is the only potentially curable option for CRS. However, gastrectomy is more difficult for CRS than for primary GC due to intra-abdominal adhesions, anatomic variation, and changes in lymphatic flow and the celiac axis caused by initial surgery [5, 6]. Emerging evidence has shown that GC patients undergoing laparoscopic gastrectomy (LG) have better short-term and comparable long-term oncological outcomes than those who undergo conventional open surgery [7–10]. However, this technology has some insuperable limitations, including limited movement of laparoscopic instruments, unavoidable physiological tremors, and long learning curve, thus hindering the promotion of LG [11, 12]. Robotic surgery has offered a new alternative surgical approach and became an alternative procedure in the treatment of GC. Theoretically, robotic gastrectomy (RG) has advantages over LG of dexterity and accuracy because of tremor filter, 3D imaging, and an internal Endo Wrist with seven degrees of freedom. Therefore, the benefits of RG might be more evident in complex cases, such as those with CRS. To date, studies have reported that laparoscopic total gastrectomy (LTG) for CRS can be a safe treatment option, but the surgical outcomes of robotic total gastrectomy (RTG) for CRS were seldom reported [3, 13, 14].

Thus, we designed this study to evaluate surgical outcomes of RTG for CRS by comparing short-term and oncological outcomes with LTG.i

## Patients and methods

### Patients

In this cohort study, we collected the data of patients who underwent RTG or LTG for CRS in Southwest Hospital (Chongqing, China) between May 2006 and October 2019. The definition of CRS is carcinoma in the remnant stomach, which does not distinguish the length of the interval period or the feature of previous disease [1]. Inclusion criteria of the study were as follows: (i) histologically confirmed adenocarcinoma by gastroscopy and pathological biopsy; (ii) depth of invasion confined to cT1 to T4a; (iii) no distant metastasis; (iv) age >18 and <80 years; (v) ASA class I, II, or III; and (vi) without neoadjuvant chemotherapy. Patient background, surgical outcomes, pathologic stage, and follow-up results were investigated. Pathologic stage was classified according to the 8th TNM classification [15]. The present study was approved by the Ethics Committee of our center (No. KY2020071).

### Surgical procedures

All surgeries were performed by one expert surgeon and the learning curve of RG was surpassed prior to RTG for CRS. The surgeon had performed 62 laparoscopic and 27 RG surgeries before initiating laparoscopic and robotic surgeries for CRS. The surgical method (robotic or laparoscopic) was selected by each patient. The da Vinci surgical system was used. The procedures for laparoscopic surgery have been previously described in detail [16, 17].

### Follow-up

The patients were regularly followed up at 3-month intervals in the first year and then at 6-month intervals thereafter. Abdominopelvic computed tomography, upper gastrointestinal endoscopy, and blood tests including serum carcinoembryonic antigen were performed for regular follow-up and detection of recurrence, which was confirmed by cytology or histology if necessary. Overall survival (OS) time was calculated from the date of operation to the date of all-cause death or the last follow-up. Disease-free survival (DFS) time was calculated from the date of operation to the date of recurrence, all-cause death, or the last follow-up. All patients were followed until death or until the last follow-up date of 31 March 2020.

### Post-operative evaluation and outcome measurements

Post-operative complications were classified according to the revised version of the Clavien–Dindo (C-D) classification system [18, 19]. Grade II complications were considered as moderate and grade IIIa or greater complications, which required additional interventional or surgical treatment, were considered severe complications. The effective operation time indicated the time required for technical steps. The total cost included all costs during hospitalization (including surgery, surgical equipment, lab tests, medicine). Additionally, costs due to post-operative complications were also taken into account.

### Statistical analysis

Continuous variables are presented as mean ± standard deviation and categorical variables are expressed as frequency and percentages. The comparisons among groups are tested with Fisher’s exact test, Student’s *t*-test, or Chi-square test. Survival curves were estimated and compared using the Kaplan–Meier method and log-rank test. *P *<* *0.05 was considered statistically significant. Statistical analysis was performed using IBM SPSS Statistics 25 (IBM Corporation, Armonk, NY, USA).

## Results

### Patient characteristics

Patient characteristics are listed in **Table 1**. The indications for initial gastrectomy, previous surgical method, previous resection extent, and previous reconstruction types were similar between the robotic and laparoscopic groups.

**Table 1.** Characteristics of patients with carcinoma in the remnant stomach

Characteristic	Robotic group (*n* = 29)	Laparoscopic group (*n* = 41)	*P*-value
Age (mean ± SD) (years)	60.3 ± 12.6	58.2 ± 9.8	0.426
Sex [*n* (%)]			0.981
Male	22 (75.9)	31 (75.6)	
Female	7 (24.1)	10 (24.4)	
BMI (mean ± SD) (kg/m^2^)	19.4 ± 2.2	20.4 ± 2.5	0.100
Original disease [*n* (%)]			0.977
Benign disease	10 (34.5)	14 (34.1)	
Cancer	19 (65.5)	27 (65.9)	
Previous surgical method [*n* (%)]			0.189
Open	25 (86.2)	36 (87.8)	
Laparoscopic	4 (13.8)	5 (12.2)	
Previous resection extent [*n* (%)]			0.771
Distal gastrectomy	28 (96.6)	39 (95.1)	
Proximal gastrectomy	1 (3.4)	2 (4.9)	
Previous reconstruction [*n* (%)]			0.926
Billroth I	3 (10.3)	5 (12.2)	
Billroth II	25 (86.2)	34 (82.9)	
Esophagogastrostomy	1 (3.4)	2 (4.9)	

SD, standard deviation; BMI, body mass index.

### Surgical outcomes

Surgical outcomes are summarized in **Table 2**. The robotic group showed similar effective operation times (272.0 vs 297.9 min, *P *=* *0.170), less estimated blood loss (229.2 vs 288.8 mL, *P *=* *0.031), and higher total costs (105,967.2 vs 81,629.5 RMB, *P *<* *0.001) as compared with the laparoscopic group. Five cases (17.2%) in the robotic group and eight cases (19.5%) in the laparoscopic group underwent conversion to open surgery. The conversion rates were similar between the two groups (*P *=* *0.810). The reasons for conversion in the robotic group included extensive adhesions (three cases), tumor invasion to the transverse colon (one case), and tumor invasion to the diaphragm (one case), whereas the reasons for conversion in the laparoscopic group included severe adhesions (fice cases), uncontrolled diffuse bleeding in the operation field (two cases), and tumor invasion to the transverse colon (one case). Compared with the laparoscopic group, the robotic group showed similar results in terms of time to first flatus (2.3 vs 2.5 days, *P *=* *0.413), time to first soft diet (4.9 vs 5.2 days, *P *=* *0.502), and post-operative hospital stay (9 vs 9 days, *P *=* *0.894).

**Table 2.** Surgical results of the robotic and laparoscopic groups

Characteristic	Robotic group (*n* = 29)	Laparoscopic group (*n* = 41)	*P*-value
Effective operation time (mean ± SD) (min)	272.0 ± 88.2	297.9 ± 68.5	0.170
Estimated blood loss (mean ± SD) (mL)	229.2 ± 88.7	288.8 ± 124.6	0.031
Conversion to open surgery [*n* (%)]	5/29 (17.2)	8/41 (19.5)	0.810
Time to first flatus (mean ± SD) (days)	2.3 ± 1.0	2.5 ± 1.0	0.413
Time to first soft diet (mean ± SD) (days)	4.9 ± 1.7	5.2 ± 2.0	0.502
Post-operative hospital stay, median [*n* (IQR)] (days)	9 (7–10)	9 (8–10)	0.894
Total cost (mean ± SD) (RMB)	105,967.2 ± 16,897.1	81,629.5 ± 17,589.9	< 0.001

SD, standard deviation; IQR, interquartile range.

### Post-operative complications and mortality

Post-operative complications and mortality are shown in **Table 3**. The overall complication rates did not differ between the robotic and laparoscopic groups (27.6% vs 22.0%, *P *=* *0.588) and there were no significant differences in moderate (13.8% vs 14.6%, *P *=* *1.000) or severe complications (13.8% vs 7.3%, *P *=* *0.627). One patient in the laparoscopic group died due to uncontrollable intra-abdominal infection. The mortality was similar between the robotic and the laparoscopic groups (0.0% vs 2.4%, *P *=* *0.861).

**Table 3.** Post-operative complications and mortality of the robotic and laparoscopic groups

Characteristic	Robotic group (*n* = 29)	Laparoscopic group (*n* = 41)	*P*-value
Overall complications [*n* (%)]	8 (27.6)	9 (22.0)	0.588
Moderate complications (C-D II) [*n* (%)]	4 (13.8)	6 (14.6)	1.000
Severe complications (C-D ≥ IIIa) [*n* (%)]	4 (13.8)	3 (7.3)	0.627
Post-operative general complications [*n* (%)]			
Wound infection	1 (3.4)	2 (4.9)	1.000
Intra-abdominal infection	1 (3.4)	2 (4.9)	1.000
Pneumonia	1 (3.4)	3 (7.3)	0.870
Post-operative surgical complications [*n* (%)]			
Esophagojejunostomy leakage	3 (10.3)	2 (4.9)	0.686
Intestinal leakage	1 (3.4)	1 (2.4)	1.000
Intra-abdominal hemorrhage	1 (3.4)	0 (0.0)	0.414
In-hospital mortality [*n* (%)]	0 (0.0)	1 (2.4)	0.861

CD grade indicates Clavien–Dindo grade.

### Pathologic and survival outcomes

Pathologic results are summarized in **Table 4**. The robotic and laparoscopic groups showed similar results in tumor location (*P = *0.467), histologic type (*P = *0.368), tumor size (*P = *0.408), rate of lymph-node metastasis (58.6% vs 53.7%, *P = *0.681), number of retrieved lymph nodes (13.6 vs 11.2, *P = *0.150), R0 resection rate (93.1% vs 90.2%, *P = *0.990), and pTNM stage (*P *=* *0.099).

**Table 4.** Pathologic results of the robotic and laparoscopic groups

Characteristic	Robotic group (*n* = 29)	Laparoscopic group (*n* = 41)	*P*-value
Tumor location [*n* (%)]			0.467
Anastomosis	13 (44.8)	22 (53.7)	
Non-anastomosis	16 (55.2)	19 (46.3)	
Histologic type [*n* (%)]			0.368
Differentiated	7 (24.1)	14 (34.1)	
Undifferentiated	22 (75.9)	27 (65.9)	
Tumor size (mean ± SD) (cm)	4.0 ± 2.1	3.7 ± 1.6	0.408
Lymph-node metastasis [*n* (%)]			0.681
No	12 (41.4)	19 (46.3)	
Yes	17 (58.6)	22 (53.7)	
No. of retrieved lymph nodes (mean ± SD)	13.6 ± 8.1	11.2 ± 5.3	0.150
No. of retrieved lymph nodes (previous benign disease)	20.1 (9.5)	17.0 (4.6)	0.297
No. of retrieved lymph nodes (previous malignant disease)	10.2 (4.7)	8.3 (2.5)	0.082
R0 resection [*n* (%)]	27 (93.1)	37 (90.2)	0.990
pTNM stage [*n* (%)]			0.099
I	2 (6.9)	11 (26.8)	
II	6 (20.7)	8 (19.5)	
III	21 (72.4)	22 (53.7)	

SD, standard deviation.

**Figure 1.**
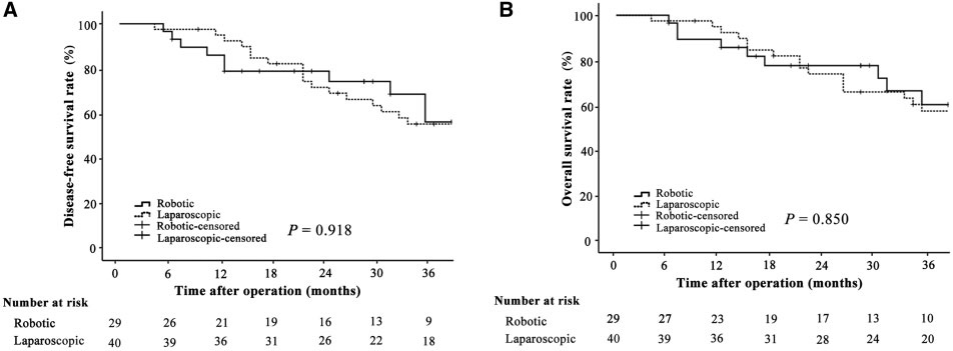
Kaplan–Meier curves of 3-year survival outcomes for robotic vs laparoscopic gastrectomy. (A) Disease-free survival rate of the robotic and laparoscopic groups; (B) overall survival rate of the robotic and laparoscopic groups.

The median follow-up periods of the robotic and laparoscopic groups were 31 months (interquartile range, 19–62 months) and 38 months (interquartile range, 23–69 months), respectively. During the follow-up period, 10 patients in the robotic group and 18 patients in the laparoscopic group experienced tumor recurrences. The recurrence patterns are summarized in **Supplemental Table 1**. The 3-year DFS rates were 65.5% in the robotic group and 57.5% in the laparoscopic group. Kaplan–Meier curves for DFS showed no significant differences between the two groups (*P *=* *0.918; **Figure 1A**). The 3-year OS rates were 69.0% in the robotic group and 60.0% in the laparoscopic group. Kaplan–Meier curves for OS showed no significant differences between the two groups (*P *=* *0.850; **Figure 1B**). The 3-year survival rates were similar in the subgroup analysis of survival outcomes based on different types of initial surgery (**Supplemental Figure 1**).

## Discussion

In recent years, LTG for the treatment of CRS has been documented in some case studies and clinical trials [5, 13, 14, 20]. Based on current evidence, laparoscopic surgery can be safely performed in patients with CRS by experienced surgeons. Our previous study demonstrated that RG is a safe and feasible surgical procedure in the treatment of GC [21–23].

Alhossaini *et al*. [24] reported that the operation time in the robotic surgery group was longer than that in laparoscopic surgery group. However, we found no difference in the effective operation time between the two groups. Extensive research has reported that patients undergoing RG had significantly longer operative times when compared with those who underwent LG [25–27]. Liu *et al.* [28] identified the reasons why RG requires more time than LG and found that the main cause of the prolonged time in robotic surgery is the junk time (instrument setup and docking or positioning of surgical arms). In our study, we found that the two groups had similar effective operation times. This indicated that the time for dissection or reconstruction was almost identical between the two groups.

A recent study reported that the robotic group had a lower conversion rate than the laparoscopic group [24]. Based on published studies, the conversion during minimally invasive surgery was mainly attributed to extensive adhesions due to previous abdominal surgery [5, 29, 30]. In clinical practice, we found that patients with a history of minimally invasive surgery always had less adhesion than those who had undergone previous open surgery. The dense adhesions may cause injuries of adjacent organs during adhesiolysis. In the present study, open surgery was the most common surgical method for initial gastrectomy. This could explain why patients in this study had a higher conversion rate than those in the study by Alhossaini *et al.* [24].

Post-operative complications is a core index for evaluating the safety and feasibility of a surgical procedure. A recent meta-analysis revealed that RG and LG had similar complication rates in the treatment of GC [31]. In the present study, the post-operative complications were similar between the two groups. Additionally, the robotic group also showed similar post-operative recovery outcomes as compared with those of the laparoscopic group. According to the present findings, it seems that optimal preoperative outcomes may have been achieved by LG and enhanced recovery after surgery programs have been routinely applied in patients who have undergone gastrectomy in our center, leaving limited room for improvement via RG. Robotic surgery for CRS seems not to be superior to laparoscopic surgery because of its longer operation time and higher costs. In addition, it is inconvenient for comprehensive abdominal exploration because the robotic arms only have a restricted range of activities once installed and cannot change direction freely. With this robotic surgical system, we cannot freely examine the whole abdomen including both the upper and lower abdomen unless we change the patient’s position. Despite these limitations, we do realize that robotic surgery for CRS has some advantages in the view of surgeons. The robotic systems provide a superior operative environment to conventional laparoscopic surgery in the management of CRS. However, the benefits of subjective feelings such as improved visibility and ease of accurate dissection are hard to quantify and compare.

A growing number of studies have reported the ergonomic benefit of robotic surgical platforms to the operating surgeon [32]. In our clinical practice, the first arm is used for main operations and an ultrasonic scalpel is usually installed to complete lymphadenectomy for radical gastrectomy in GC. But unlike radical gastrectomy for GC, the key difficulty in the operation for CRS is to loosen and separate the abdominal adhesions; there are no major blood vessels in the dense fiber tissue. Therefore, we suggest a flexible electric hook with more degrees of freedom instead of an ultrasonic scalpel that cannot rotate. The second arm is mainly used to cooperate with the first arm for completing delicate manipulations and we configure small and dexterous grasping forceps for slightly clamping the lymph adipose tissue and membrane structure. The third arm is mainly used for lifting or suspending the intestine and the liver to expose the visual field, so its fixed time is much longer than its free time. We chose two-hole grasping forceps that had a larger contact area in order to reduce damage to the intestinal wall. Moreover, this rotatable forceps’ effect of lifting or suspending the intestine or the liver is much better than that of the assistant in laparoscopic surgery because it can form a stereoscopic structure when opened. Meanwhile, it is stable and tireless when locked up. Using the three arms, the surgeon can perform the operation more easily with less dependence on the assistant. This superiority is very important during the surgical procedure, because the surgery for CRS often takes a long time and it is tiring for the assistant to lift or suspend the intestine and the liver for such a long time. Additionally, the surgeon can keep a comfortable sitting position during robotic surgery and therefore can focus more on the operation.

We have reported that LTG for CRS has particular advantages over open surgery, including better exposure of the surgical visual field, easier abdominal adhesiolysis, and more convenient operation in a narrow space [33]. The robotic system expands these advantages by its 3D imaging and improves dexterity resulting from an internal articulated EndoWrist that allows seven degrees of freedom. Moreover, under the stereoscopic vision, we can strip the vascular along the tunica adventitia and dissect the lymphatic tissue more precisely.

In the present study, although the 3-year survival rates showed no significant difference between the robotic and laparoscopic groups, the relatively small sample size made it difficult to generalize the findings. Additionally, we observed a tendency favoring the robotic group. These null results should be interpreted with caution because the statistical power may have been more sufficient if the study had been designed with a larger sample size and longer follow-up period.

Our study had several limitations. First, there was potential selection bias due to its retrospective design. Second, the sample size of each group was relatively small. In addition, this study was conducted in a high-volume center and the surgeon was an expert in this area, which may have limited the general applicability of these results.

In conclusion, this study suggests that RTG is a safe and feasible procedure for CRS and could serve as an optimal treatment for CRS. Robotic systems provide a superior operative environment that could help surgeons to carry out technically demanding operations more easily and comfortably. Future properly designed large-scale randomized–controlled trials are needed to confirm these results.

## Supplementary Data

Supplementary data is available at *Gastroenterology Report* online.

## Authors’ Contributions

Z.Y.L. and F.Q. designed the study. Z.Y.L., J.J.L., B.W., and J.J.W. were responsible for data acquisition. P.W.Y., Y.L.Z., and Y.S. were responsible for quality control of the data and algorithms analysed, and interpreted the data. Z.Y.L. and J.J.L. wrote the article. All authors read and approved the final manuscript.

## Funding

None.

## Supplementary Material

goab021_supplementary_data
