# Supplementary figures and images for: Robotic total gastrectomy for carcinoma in the remnant stomach: a comparison with laparoscopic total gastrectomy
Source: Gastroenterol Rep (Oxf). 2021 Jul 19;9(6):583–8. doi: 10.1093/gastro/goab021 (PMC8677512; doi:10.1093/gastro/goab021)

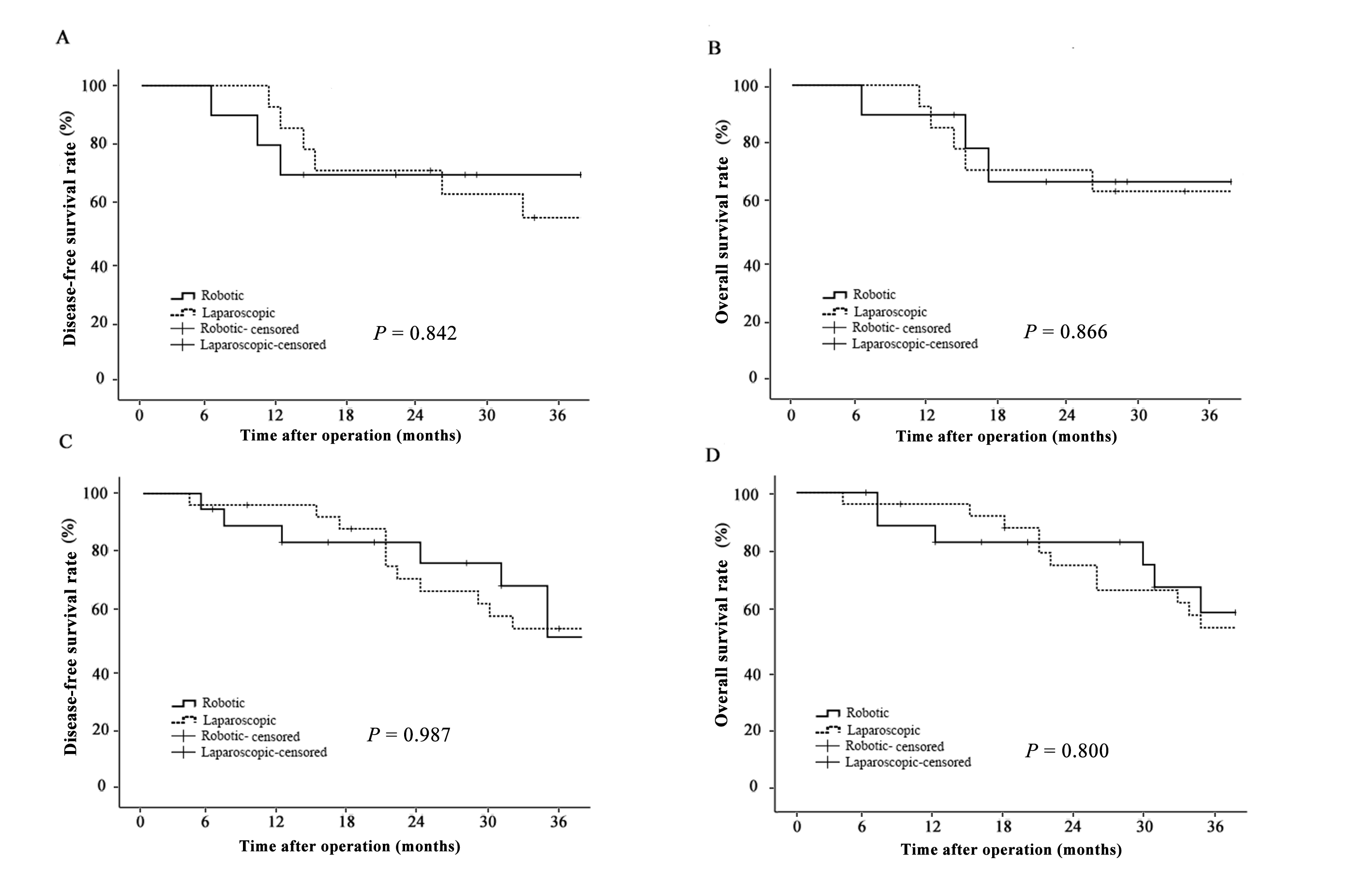

Supplement: goab021_supplementary_data [file goab021_supplementary_data.zip › goab021_Supplementary_Data.tif]
